# PLGA Microparticles as a Stable and Biocompatible Carrier for Adiponectin Delivery to Enhance Bone Regeneration

**DOI:** 10.3390/pharmaceutics18050546

**Published:** 2026-04-29

**Authors:** Pengxin Zhang, Yang Wang, Fan Hu, Yanping Gong

**Affiliations:** 1 Department of Endocrinology, The Second Medical Center, The Chinese People’s Liberation Army General Hospital, Beijing 100853, China; pengxinzh@163.com (P.Z.); yangwang19880717@163.com (Y.W.); hufan@301hospital.com.cn (F.H.); 2 National Clinical Medical Research Center for Geriatric Diseases, The Chinese People’s Liberation Army General Hospital, Beijing 100853, China; 3 Graduate School of the Chinese People’s Liberation Army General Hospital, Beijing 100853, China

**Keywords:** adiponectin, PLGA, microparticles, biocompatibility, bone regeneration

## Abstract

**Background**: Adiponectin (ADPN) is a key adipokine with osteogenic potential, but its clinical translation for bone regeneration is hindered by poor in vivo stability. This study aimed to develop poly lactic-co-glycolic acid (PLGA) microparticles as a stable and biocompatible carrier for sustained ADPN delivery to enhance bone repair. **Methods**: ADPN-loaded PLGA microparticles (ADPN-MPs) were fabricated via emulsion solvent evaporation. Their physicochemical properties were characterized using scanning electron microscopy (SEM) and circular dichroism (CD) spectroscopy. Loading efficiency and drug loading were quantified. In vitro release kinetics and stability under physiological conditions were assessed. Biocompatibility was evaluated using MC3T3-E1 osteoblasts and BMSCs, and in vivo efficacy was tested in a fracture model via gait analysis. **Results**: Employing CD to evaluate the secondary structure of ADPN, emulsion solvent evaporation for microparticles preparation, and SEM for morphological analysis, we quantitatively assessed the loading efficiency (69.83 ± 4.24%) and drug loading (0.97 ± 0.06%) of ADPN-MPs. Results indicated that ADPN-MPs maintained significant stability under varied pH and temperature conditions and exhibited a controlled release profile, with an average initial rapid release of 14.25% within 24 h and an average cumulative release of 55.00% by day 28. Furthermore, ADPN-MPs promoted the proliferation of MC3T3-E1 and BMSCs without toxicity, demonstrating excellent biocompatibility. Notably, gait analysis in a fracture model showed improved healing in both ADPN and ADPN-MPs groups compared to controls, with ADPN-MPs demonstrating comparable efficacy to free ADPN, supporting its potential as a stable delivery system for bone regeneration. **Conclusions**: PLGA microparticles serve as an effective, stable, and biocompatible delivery platform for ADPN, significantly promoting bone regeneration in vitro and in vivo. This delivery system enhances the therapeutic potential of ADPN for clinical bone repair applications.

## 1. Introduction

Epidemiologically, fractures represent a major and growing clinical challenge, with millions of incidents annually leading to prolonged disability, elevated mortality, and substantial healthcare expenditures worldwide [1]. The fracture healing process is often impaired in aged or osteoporotic bone due to a compromised regenerative microenvironment, resulting in delayed union or non-union. Current clinical strategies primarily provide mechanical stabilization or systemic anti-resorptive therapy, yet they offer limited efficacy in actively overcoming the local biological deficits that hinder robust bone repair [2,3]. Therefore, there is a pressing need for novel interventions that can precisely modulate the molecular pathways governing osteogenesis to achieve timely and effective fracture healing. Recent advances in biomaterials engineering have enabled the development of smart scaffolds that deliver osteo-inductive growth factors, microRNAs, or small-molecule drugs—thereby enhancing endogenous regenerative capacity while minimizing off-target effects [4,5].

Adiponectin (ADPN), a multifunctional adipokine primarily secreted by adipose tissue, plays a crucial role in metabolic regulation and significantly influences bone metabolism [6]. Studies have demonstrated ADPN’s ability to enhance osteoblast proliferation and differentiation, thereby promoting bone regeneration and increasing bone mineral density [7]. Specifically, ADPN has been shown to stimulate osteoblast differentiation through activation of the AMP-activated protein kinase (AMPK) signaling pathway and upregulation of osteogenic markers such as Runx2 and alkaline phosphatase [8]. Additionally, ADPN inhibits osteoclastogenesis by suppressing receptor activator of nuclear factor kappa-Β ligand (RANKL)-induced NF-κB signaling, thus reducing bone resorption [9]. These dual effects contribute to the regulation of the bone microenvironment and maintenance of bone homeostasis [10]. These findings highlight ADPN’s potential in accelerating fracture healing. However, despite its promising therapeutic effects, the clinical application of ADPN is limited by its instability and rapid degradation in physiological environments, which reduces its bioavailability and efficacy.

Recent studies on ADPN sustained-release formulations have explored various delivery systems such as hydrogels, liposomes, and polymeric nanoparticles to improve the stability and bioavailability of ADPN for therapy [11,12]. Hydrogels provide a favorable environment for protein loading and sustained release, but are limited by weak mechanical strength and an initial burst release. Liposomes offer good biocompatibility and efficient cellular uptake, yet face challenges in stability and large-scale production [13]. Conversely, polymeric microparticles, specifically poly lactic-co-glycolic acid (PLGA), involve loading the protein into porous PLGA microparticles through physical dispersion. It highlights its potential to overcome the limitations of other systems [14]. Given PLGA’s biodegradability and biocompatibility approval by the FDA (U.S. Food and Drug Administration) and the CDE (Center for Drug Evaluation, China), it is an ideal candidate for developing ADPN sustained-release formulations [15]. Therefore, investigating PLGA-based microcarriers is essential to create effective and safe ADPN delivery systems, with promising applications such as promoting bone growth in fracture treatment [16]. This strategy is implemented via self-healing post-loading technology, which uses heat to seal polymer pores and load proteins without organic solvents [17]. However, while these conditions facilitate microparticle sealing, they are generally unfavorable for maintaining protein bioactivity—a particular concern for ADPN, whose conformation is highly sensitive to thermal stress [18].

This prompted us to explore a modified self-healing approach using reduced-temperature conditions tailored to ADPN’s thermosensitive nature, achieving effective microparticle sealing without compromising protein integrity—a strategy that preserves bioactivity, overcomes the stability limitations of free ADPN, and thereby advances its potential as a therapeutic strategy for enhancing bone fracture healing.

## 2. Materials and Methods

### 2.1. Stability of ADPN

The ADPN raw material, synthesized by Leo Bio-technology Co., Ltd., Xuzhou, China, was formulated in 10 mM phosphate buffer containing 150 mM NaCl, 5–7% (*w*/*v*) mannitol, and 0.005% (*v*/*v*) Tween 80, with the final pH adjusted to 6.5–7.2. The secondary structure of ADPN was assessed using circular dichroism (CD) spectroscopy (JASCO, Tokyo, Japan) under varying temperatures and pH levels. For this analysis, ADPN was dissolved in water to obtain a concentration of 0.2 mg/mL. The ADPN solution was then incubated under the following conditions: 4 °C for 18 h (Control), 37 °C for 18 h, and 45 °C for 12 h. To assess pH stability, the 0.2 mg/mL ADPN solution was adjusted to pH 3 and pH 5 by adding dilute phosphate and incubated at 4 °C for 18 h. All samples were subsequently analyzed by CD spectroscopy. Each measurement was performed in triplicate, and the average value was recorded. Spectra were acquired over the wavelength range of 190–400 nm with a step interval of 0.5 nm.

### 2.2. Preparation of ADPN-MPs

PLGA (acid-terminated, lactide:glycolide ratio = 75:25, Mw = 8–14 kDa) was purchased from Evonik Industries, Essen, Germany. The PLGA porous microparticles were prepared using the emulsion solvent evaporation method [19]. Specifically, a primary emulsion was first formed by dissolving 400 mg of PLGA in 2 mL of dichloromethane (Sinopharm Group Chemical Reagent Co., Ltd., Shanghai, China), followed by the addition of 0.5 mL of 0.5% (*w*/*v*) aqueous sodium bicarbonate solution (Sinopharm Group Chemical Reagent Co., Ltd., Shanghai, China) and homogenization at 12,000 rpm for 2 min. The resulting primary emulsion was then transferred into 30 mL of 1% (*w*/*v*) aqueous poly(vinyl alcohol) (PVA) solution (Merck KGaA, Darmstadt, Germany) and homogenized at 6200 rpm for 1.5 min using a T18 basic homogenizer (IKA, Staufen, Germany) to form a double emulsion. The double emulsion was subsequently poured into 500 mL of deionized water and stirred at 300 rpm for 4 h to facilitate dichloromethane evaporation. After three washes with distilled water, the porous microparticles were collected. After preparation, they were freeze-dried and resuspended in purified water. The supernatant was removed following centrifugation. ADPN powder was dissolved in purified water, and 4 mL of a 0.2 mg/mL ADPN solution was added to the PLGA microparticles suspension. The mixture was then placed in a shaker at 4 °C for 12 h, followed by transfer to a water bath shaker at 37 °C, operating at 100 rpm for an additional 18 h. ADPN diffuses into the pre-formed porous PLGA microparticles, and upon completion of the self-healing process, a certain amount of ADPN is also adsorbed onto the surface of the microparticles.

### 2.3. Observation and Analysis of Surface Pore Diameter of Microparticles Utilizing Scanning Electron Microscopy

The PLGA suspension was thoroughly mixed, and 10 µL of the suspension was deposited onto a silicon wafer. After air-drying at ambient temperature, the sample was sputter-coated with gold. The accelerating voltage and working distance were not fixed and were adjusted in real-time during imaging to achieve optimal image clarity. The surface morphology of the microparticles was observed using a JSM7900F scanning electron microscope (SEM) (Hitachi Instruments Inc., Hitachi, Japan).

### 2.4. Drug Loading Determination Experiment

The prepared self-healing microparticles were centrifuged at 4000× *g* for 2 min using a high-speed centrifuge, and the supernatant was collected. To evaluate free ADPN protein in the supernatant before and after loading, SDS-PAGE electrophoresis was first performed to qualitatively assess the integrity and purity of ADPN. For this analysis, ADPN was dissolved in water at a concentration of 0.2 mg/mL. Samples collected from the initial ADPN solution and from the supernatant after the PLGA loading procedure were both subjected to SDS-PAGE. The amount of free ADPN was then quantified using the Bicinchoninic Acid (BCA) (Thermo Fisher Scientific, Waltham, MA, USA) protein assay at 562 nm, which measures total protein concentration. Under our experimental conditions, where ADPN is the predominant protein in the supernatant, total protein content reliably reflects ADPN levels. The equations for drug loading (DL) and loading efficiency (LE) are as follows:

(1) $$DL\left(\%\right)=\left(\frac{Weight\;of\;drug\;entrapped}{Weight\;of\;microparticles+Weight\;of\;drug\;entrapped}\right)\times 100$$

(2) $$LE\left(\%\right)=\left(\frac{Loaded\;drug}{Total\;drug}\right)\times 100$$

### 2.5. Measurement of Particle Size

The particle size of the porous microparticles was analyzed using a micrometer particle diameter analyzer (HELOSBR, Sympatec GmbH, Remlingen, Germany), with the particle size determined via the laser diffraction method. The instrument’s dispersion pressure was set at 3 bar, and the feed concentration exceeded 2%. D_10_, D_50_, D_90_, and span values of the microparticles were recorded to ascertain their size and distribution. The calculation equation for the distribution span of the microparticle size is as follows:

(3) $$Span=\frac{{(D}_{90}-D_{10})}{D_{50}}$$

### 2.6. In Vitro Release of ADPN

After loading ADPN into the microparticles, the release profile was evaluated in 0.01 mol·L^−1^ PBS (pH 7.4) as the release medium (3 mL per sample). The system was maintained at 37 °C with linear shaking at 120 rpm. Samples were collected at predetermined intervals (0, 1, 3, 7, 14, 21, and 28 days), withdrawing 3 mL of supernatant each time and replacing it with fresh PBS (Beijing Solarbio Science & Technology Co., Ltd., Beijing, China). The morphological characteristics of the microparticles were examined by SEM at selected time points to assess structural changes during release. The amount of released ADPN was measured at 562 nm using a BCA protein assay kit. Additionally, the cumulative release percentage was calculated as the ratio of released ADPN at each time point to the total loaded ADPN.

### 2.7. Biocompatibility Testing of ADPN-MPs

ADPN microparticles were added to the culture solution to prepare a medium with a concentration of 10 μg/mL ADPN protein according to our previously published research [20]. Monolayer adherent Mouse Calvaria Osteoblast Precursor Cells (MC3T3-E1) (ATCC^®^ CRL-2593™, passage 5) in the logarithmic growth phase were seeded into a 96-well plate at a density of 5 × 10^3^ cells per well and incubated for 1, 4, and 7 days. Untreated cells (without ADPN microparticles) served as controls. Subsequently, 10 μL of CCK-8 solution was added to each well, and the cells were further incubated in a cell culture incubator for 2 h. Absorbance values were measured using a multimode microplate reader (Varioskan LUX, Thermo Fisher Scientific, Waltham, MA, USA) at a detection wavelength of 450 nm. Details are described in Supplementary Table S1.

Bone marrow-derived mesenchymal stem cells (BMSCs) were isolated from 3-day-old Sprague-Dawley rat pups. For experiments, passage 3 BMSCs were used. 200 μL of BMSCs suspension was seeded into a 96-well plate at a density of 2 × 10^3^ cells per well and incubated in a constant temperature cell incubator for 1, 4, and 7 days. Untreated cells served as controls. Under light-protected conditions, 20 μL of 5 mg/mL MTT solution was added to each well and incubated for 4 h in the constant-temperature cell incubator. Subsequently, 150 μL of DMSO was added to each well, and the absorbance values were measured at a wavelength of 490 nm using a multimode microplate reader. Details are described in Supplementary Table S2.

### 2.8. Biomechanical Testing of ADPN-MPs

A total of 12 specific-pathogen-free C57BL/6 mice (male, aged 8 weeks, weighing 18–22 g) were randomly divided into three groups: control (PBS), free ADPN, and ADPN-MPs (*n* = 4 per group). A tibial fracture model was established on both sides under general anesthesia (2% pentobarbital sodium, 60 mg/kg, i.p.) in the middle using a bone cutter. Immediately after fracture creation, treatments were administered locally at the defect site. For the ADPN group, 1 mg/kg of ADPN was injected directly into the defect. For the ADPN-MPs group, 1 mg/kg of ADPN loaded into ADPN-MPs was injected. The control group received 0.5 mL of PBS alone. The Catwalk system (Noldus Information Technology, Wageningen, The Netherlands) was used to evaluate motor deficits and gait changes caused by pain in rodents during natural walking. The system uses footprint light refraction technology to capture the true footprints of mice through a high-speed camera placed under the walking platform, thereby measuring the relative pressure difference in footprints. Mice underwent gait analysis at weeks 2, 3, 4, 5, and 6, respectively, post-surgery to analyze the size of their footprints.

Subsequently, at week 6 post-surgery, the mice were sacrificed, and their complete tibias were obtained for Micro-CT examination using a Quantum GX μCT System (PerkinElmer, Waltham, MA, USA) with 90 kV, 80 μA, and 4.5 μm resolution. Visualization and reconstruction were performed using Quantum GX μCT Workstation software. The following parameters were analyzed: bone mineral density (BMD), bone volume/total volume (BV/TV), trabecular thickness (Tb.Th), trabecular number (Tb.N), trabecular separation (Tb.Sp), and cortical thickness (Ct.Th). A three-point bending test was then performed using a biomechanics machine (MTS 858, MTS, Eden Prairie, MN, USA) with a preload of 5 N and a constant loading rate of 5 mm/min, recording the maximum mechanical strength and load at the time of bone fracture. The three-point bending test was employed to evaluate the biomechanical properties of the healed bone.

### 2.9. Statistical Analysis

Data were processed and statistically analyzed using Excel and Graphpad Prism 10.1.2, and are presented as mean ± standard deviation (x ± s). One-way or two-way ANOVA analysis was used to compare the three groups, followed by Tukey’s post hoc test for multiple comparisons. The significance levels were defined as follows: * *p*-values < 0.05, ** *p*-values < 0.01, *** *p*-values < 0.001, **** *p*-values < 0.0001.

## 3. Results

### 3.1. The pH and Temperature Stability of ADPN

**Figure 1:**
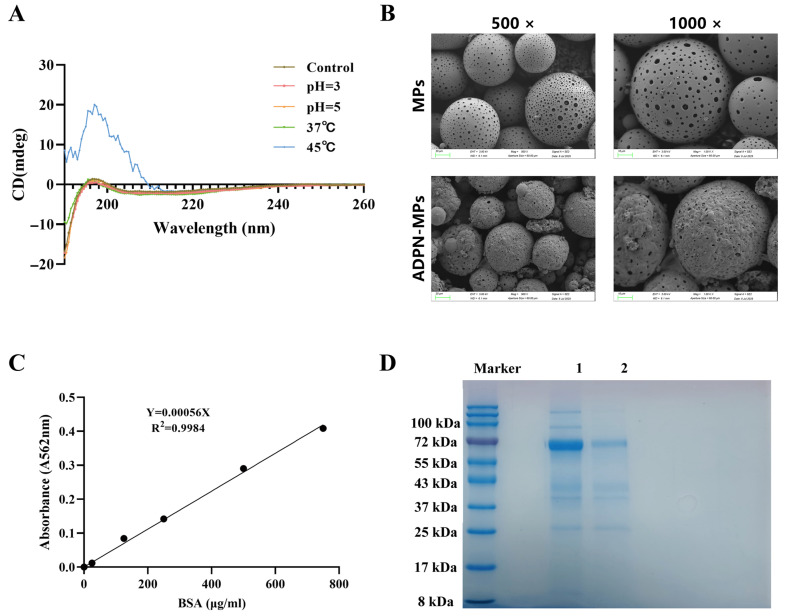
Preparation and Characterization of porous microparticles (MPs) and adiponectin self-healing microparticles (ADPN-MPs). (**A**) Analysis of PH and temperature stability of ADPN using Circular Dichroism (CD) spectroscopy; (**B**) Comparison of ADPN-MPs before and after healing under electron microscopy (scale bar = 10 μm); (**C**) Measurement of the standard curve of ADPN-MPs using the BCA method; (**D**) SDS-PAGE images of ADPN-MPs before and after loading ADPN. (1: ADPN before drug loading, 2: ADPN after drug loading).

The results of CD spectroscopy (Figure 1A) indicated that ADPN exhibits a stable secondary structure at pH 3 and pH 5, as well as at a temperature of 37 °C. However, the secondary structure of ADPN was disrupted when the temperature exceeded 45 °C. Therefore, ADPN demonstrates relatively good stability in different pH solutions, while its stability is comparatively poor at varying temperatures.

### 3.2. ADPN-MPs Have Been Successfully Fabricated

The porous microparticles were prepared using the emulsion solvent evaporation method. The surface of the completed porous microparticles is loose and porous. At 4 °C, ADPN was loaded into the MPs, followed by self-healing after the loading process was completed. Previous research has shown that morphological changes over different time points indicate that as heating time increases, particle pores significantly decrease [21]. Based on the CD spectroscopy results, we selected 37 °C for the self-healing process. After 18 h, the ADPN microparticles successfully completed self-healing without damaging the ADPN protein. A comparison of the microparticles’ morphology before and after self-healing, as observed under SEM, revealed a significant decrease in the pores on the surface of the ADPN-MPs, indicating effective loading (Figure 1B).

### 3.3. Drug Loading Capacity and Loading Efficiency

The BCA method measured the concentration of ADPN (Figure 1C), and the drug loading and loading efficiency of the ADPN-MPs were determined to be 0.97 ± 0.06% and 69.83 ± 4.24%, respectively (Table 1). The SDS-PAGE image also indicated that after loading ADPN, the concentration of ADPN in the supernatant decreased, and the band became lighter (Figure 1D), suggesting successful loading of ADPN into the microparticles while maintaining its integrity.

### 3.4. Material Advantages of ADPN-MPs

**Figure 2:**
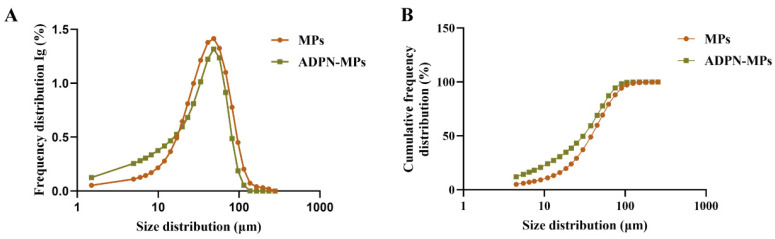
The particle size and distribution of MPs and ADPN-MPs. (**A**) The particle size frequency distribution of ADPN-MPs was measured by a micrometer particle size analyzer; (**B**) The particle size cumulative frequency distribution of ADPN-MPs was measured by a micrometer particle size analyzer.

The average particle diameter of the measured ADPN-MPs was 30.35 μm, with a span value of 2.09 μm (Table 2). Comparing the MPs and ADPN-MPs, we observed a decrease in particle size, particularly with D_90_ decreasing from 79.86 μm to 67.29 μm. This reduction in particle size will facilitate better injectability during drug administration (Figure 2). Upon the addition of purified water, the ADPN-MPs did not form crystals or small precipitates, presenting as a suspension.

### 3.5. In Vitro Release Behavior and Morphological Changes in ADPN-MPs

**Figure 3:**
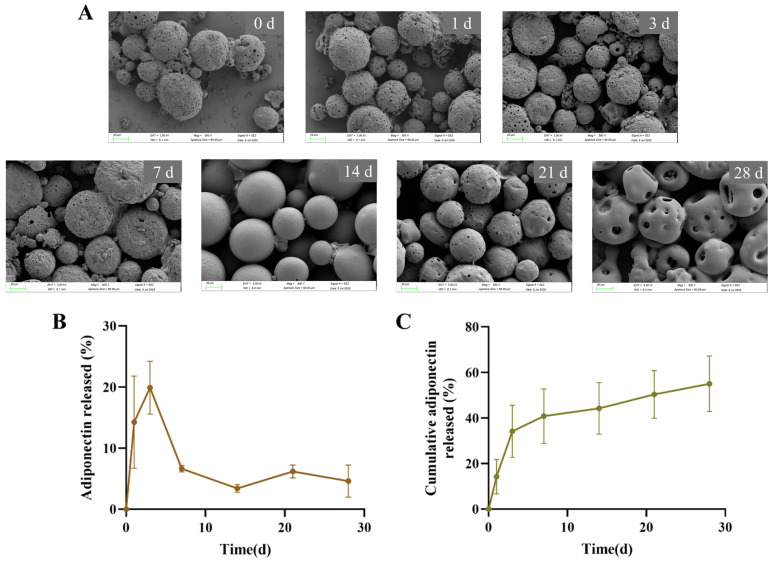
The release of adiponectin in ADPN-MPs. (**A**) SEM morphological changes in ADPN-MPs from 0 to 28 days (scale bar = 20 μm); (**B**) The release of adiponectin in ADPN-MPs from 0 to 28 days; (**C**) The cumulative release of adiponectin in ADPN-MPs from 0 to 28 days.

The morphological evolution of ADPN-MPs during in vitro release was examined by SEM. Within the first 7 days, the microparticles underwent gradual structural disintegration, exhibiting deformation, collapse, and increased surface irregularity. By day 14, polymer chain rearrangement led to partial structural reorganization, resulting in smoother and more spherical particles, and a noticeable reduction in particle size. At day 21, newly formed pores emerged on the particle surfaces, which further expanded by day 28 (Figure 3A). These observations suggest that continuous polymer degradation and chain reorganization contribute to progressive particle shrinkage, while the dynamic formation and disappearance of surface pores play a critical role in modulating ADPN release.

To quantify ADPN release kinetics, we measured the protein concentration in the supernatant over 28 days (Figure 3B). ADPN-MPs exhibited an initial burst release (14.25 ± 7.56% within the first 24 h, 19.92 ± 4.33% on days 2–3), followed by a sustained release phase on days 7–28. Subsequently, the release rate stabilized at 2–8% per week, culminating in a cumulative release of 55.00 ± 12.18% by day 28 (Figure 3C). The remaining ADPN is expected to be fully released upon complete particle degradation.

### 3.6. Biocompatibility of ADPN-MPs

**Figure 4:**
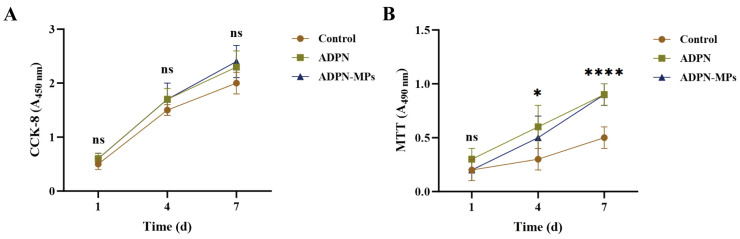
Evaluate the biocompatibility of ADPN-MPs with bone cells. (**A**) The CCK-8 method to evaluate the biocompatibility of ADPN-MPs with MC3T3-E1 cells; (**B**) The MTT method to evaluate the biocompatibility of ADPN-MPs with BMSCs. (Comparison among the three groups, * *p* < 0.05, **** *p* < 0.0001, ns means not significant).

The biocompatibility test examined the impact of ADPN-MPs on two types of bone precursor cells. The safety profile of the ADPN and ADPN-MPs group was comparable to that of the control group. The CCK-8 assay results for MC3T3-E1 cells indicated that ADPN-MPs were non-toxic and exhibited a trend towards promoting cell proliferation (Figure 4A); the MTT assay results for BMSCs revealed that both ADPN and ADPN-MPs promoted cell proliferation relative to the control group (Figure 4B). On one hand, due to the rapid cell growth, the observation of proliferative differences at 4 days was less apparent; on the other hand, the cell proliferation was almost full at 7 days, and the less difference between ADPN and ADPN-MPs group also indicates the microparticle formulation does not compromise the bioactivity of ADPN and maintains its pro-proliferative effect on these cells.

### 3.7. Biomechanical of ADPN-MPs

**Figure 5:**
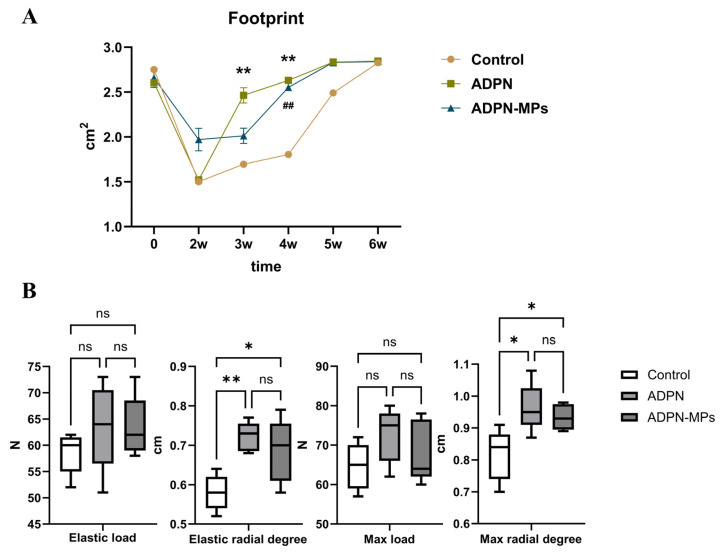
Biomechanical characteristics of ADPN-MPs. (**A**) Footprint area of mice in different groups was compared by Catwalk: the ADPN and ADPN-MPs treatment group had a larger footprint area (Comparison between control and ADPN, ** *p* < 0.01; comparison between control and ADPN-MPs, ^##^ *p* < 0.01); (**B**) The strength of callus after healing was compared by three-point bending test: the strength and toughness of ADPN and ADPN-MPs treatment group were better, especially the ADPN-MPs treatment group (* *p* < 0.05, ** *p* < 0.01, ns means not significant).

At 2 weeks post-tibial fracture surgery, the ADPN-MPs group exhibited the largest print area; by week 3, the ADPN group showed a significant increase with marked differences compared to the other two groups; by week 4, the ADPN-MPs group continuously increased to that of the ADPN group, with both groups demonstrating significant differences from the control group. The control group only reached the intervention group’s level by week 6. These findings indicate accelerated healing in the intervention groups, while the ADPN-MPs group exhibited more stable healing rates compared to the ADPN group (Figure 5A).

Although the intervention group showed no significant differences from the control group in terms of maximum load and elastic load, there were significant differences in maximum load degree and elastic load degree, indicating markedly enhanced elasticity in the intervention group. The ADPN-MPs group performed slightly better than ADPN, but the difference was not statistically significant (Figure 5B).

**Figure 6:**
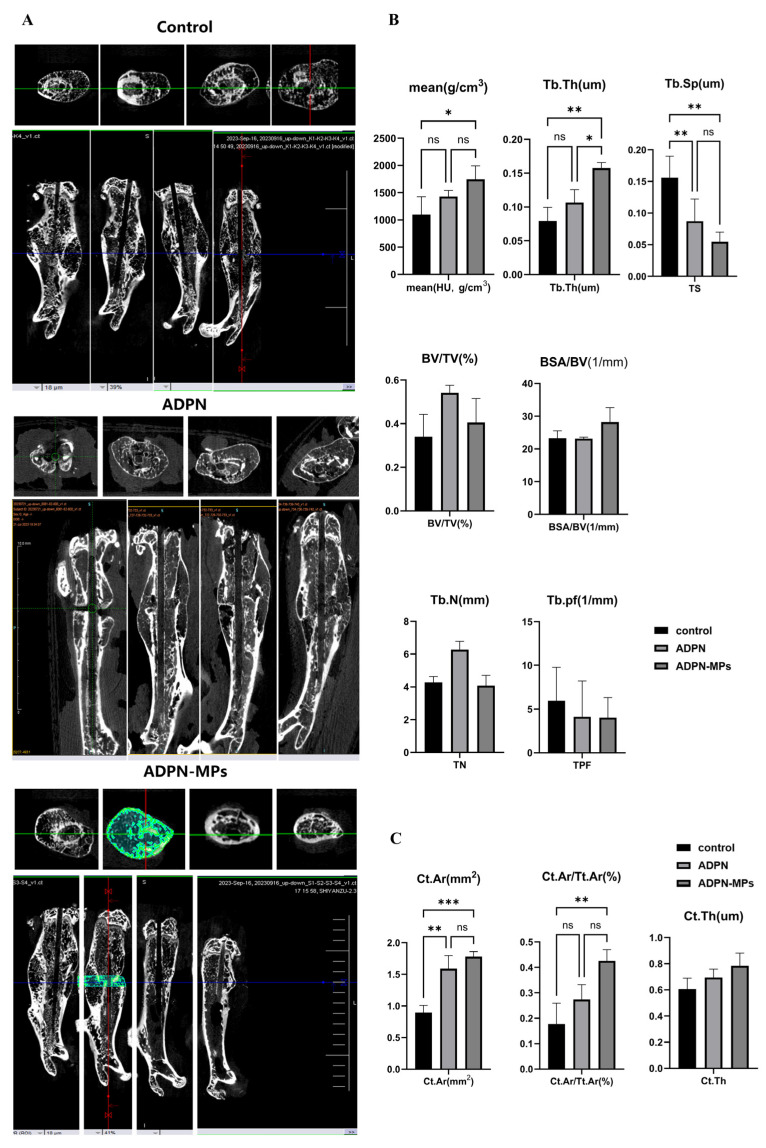
Micro-CT comparison of bone density, bone mass, trabecular and cortical bone mechanics. (**A**) The results of Micro-CT for three groups; (**B**) Trabecular bone indicators measured by Micro-CT: compared with the control group, the ADPN and ADPN-MPs treatment group can significantly improve bone density, trabecular thickness, and reduce trabecular space, with the ADPN-MPs group showing better effects; (**C**) The cortical bone indicators measured by Micro-CT: compared with the control group, the ADPN and ADPN-MPs treatment group can significantly increase the area and thickness of cortical bone, but there is no significant statistical different, the ADPN-MPs group is better than that of ADPN group. (Note: Tb.Th: trabecular thickness; Tb.Sp: trabecular space; BV/TV: bone volume/total volume; BSA/BV: bone surface area/bone volume; Tb.N: trabecular number; Tb.pf: trabecular pattern factor; Ct.Ar: cortical area; Tt.Ar: total area; Ct.Th: cortical thickness). (* *p* < 0.05, ** *p* < 0.01,*** *p* < 0.001, ns means not significant).

Compared with the control group, bone healing in the ADPN and ADPN-MPs groups occurred significantly earlier (Figure 6A). The average bone density and trabecular thickness in these groups were improved, with smaller trabecular spaces (Figure 6B), all showing statistically significant differences. The ADPN-MPs group demonstrated superiority over the ADPN group, particularly in trabecular thickness.

Compared with the control group, treatment with ADPN and ADPN-MPs significantly increased the cortical bone area (Figure 6C). The cortical bone thickness also increased, but no significant differences were observed among the three groups. The ADPN-MPs group performed better than the ADPN group, but the difference was not statistically significant.

## 4. Discussion

While PLGA-based carriers are well-established for the sustained delivery of growth factors in bone tissue engineering, their application has been predominantly focused on molecules like BMPs. The novelty of the present system lies in its targeted adaptation to overcome the specific delivery challenges of adiponectin (ADPN)—a pleiotropic metabolic regulator with a distinct, context-dependent mechanism in bone repair. Unlike canonical osteo-inductive factors, ADPN not only promotes osteogenesis but also modulates local inflammation and adipogenic-osteogenic balance, a profile that demands precise release kinetics to coordinate these phased actions. Conventional PLGA platforms typically require microparticle sealing temperatures that exceed ADPN’s conformational requirements and are often optimized for burst release or simple sustained profiles, which may not suffice. This study advances the field by demonstrating that a mild self-healing post-loading technique enables efficient loading of ADPN into PLGA microparticles, critically preserving the protein’s structure and bioactivity, achieves a release profile that aligns with the early to middle stages of fracture healing, and exerts a more effective role in promoting fracture healing. This approach thus transitions PLGA from a generic delivery vehicle to a specifically engineered system for complex metabolic mediators, offering a targeted strategy to enhance bone regeneration in compromised microenvironments such as osteoporotic fractures.

Compared with conventional PLGA platforms, the PLGA-based carriers for ADPN in this study demonstrated innovations primarily in the following two aspects. First, to optimize the loading process for this specific biologic (ADPN), we employed a mild post-loading and self-healing technique [22]. This self-healing technology leverages the segmental motion characteristics of polymers at their glass transition temperature (*T*_g_). When the external temperature exceeds the *T*_g_ of the polymer, it exhibits self-healing effects. However, our former studies have explored that in aqueous solutions, due to the plasticization effect, the polymer can undergo self-healing even when the external temperature is below its *T*_g_ [23]. By incorporating water as a plasticizer, we achieved a glass transition temperature of 37 °C. Additionally, this approach involves adsorbing the protein into pre-formed porous PLGA microparticles under mild conditions, followed by a controlled temperature-triggered ‘self-healing’ step. This method is designed to minimize the exposure of ADPN to organic solvents or harsh interfacial stresses commonly encountered during conventional emulsion-based encapsulation [22]. By carefully selecting the self-healing temperature and duration, we achieved a balance between promoting polymer chain mobility for effective pore closure and preserving the conformational integrity and bioactivity of ADPN.

The loading of ADPN within PLGA microparticles demonstrated a high drug-loading capacity and loading efficiency, as confirmed by CD, BCA assay, and SDS-PAGE analysis. These results suggest that the structural integrity of ADPN is preserved during loading, which is critical for maintaining its bioactivity. This finding aligns with previous studies indicating that protein stability within polymeric carriers largely depends on the physicochemical interactions between the protein and the polymer matrix, as well as the loading method employed [24,25]. The emulsion solvent evaporation technique used here likely minimized protein denaturation, in contrast to other methods that have shown reduced loading efficiency due to harsh processing conditions [26]. Moreover, the relatively high loading efficiency observed surpasses those reported for other adipokine delivery systems, potentially attributable to optimized polymer-to-protein ratios and emulsifier concentrations, which warrants further mechanistic exploration. This enhanced loading may directly affect therapeutic efficacy by ensuring sustained ADPN bioavailability at target sites. Thus, our approach provides a refined method for preserving ADPN stability within biodegradable carriers and produces microparticles with uniform size (~30 μm, reduced D90). This uniform size distribution enhances injectability, promotes local retention at bone defect sites, and minimizes batch-to-batch variability—factors critical for clinical translation in bone tissue engineering.

ADPN-MPs exhibited favorable biocompatibility, with a trend toward increased MC3T3-E1 proliferation and significant promotion of BMSC proliferation, indicating that the loaded ADPN retains its functional activity without compromise from the PLGA carrier. This concurs with mechanistic insights from AdipoRon studies, where activation of ADPN receptors modulated osteogenic differentiation and cellular migration through pathways including AMPK and Wnt/β-catenin [27,28]. Notably, our findings extend the understanding by demonstrating that the microparticle delivery system does not impair ADPN’s proliferative effects, which contrasts with reports where free ADPN exhibited inconsistent effects due to rapid degradation or receptor desensitization [29]. The sustained release from PLGA microparticles likely provides a more stable microenvironment, facilitating prolonged receptor engagement and downstream signaling. Additionally, the absence of toxicity suggests that the carrier material and degradation products do not adversely affect cell viability, aligning with prior evidence of PLGA’s biocompatibility in orthopedic applications [26]. These results suggest that PLGA microparticles can serve as a biocompatible carrier for ADPN, highlighting the potential of such delivery systems to support sustained bioactivity in bone regeneration applications.

A comparison of release at different time points reveals considerable intra-group variability at Day 1 and Day 3, whereas variability from Day 7 to Day 28 is relatively minor. This phenomenon may be attributed to the fact that the release at Day 1 and Day 3 corresponds to ADPN adsorbed on the microparticle surface, while the subsequent release from Day 7 to Day 28 is associated with ADPN encapsulated within the microparticles and released upon polymer degradation. The biphasic release profile of ADPN from PLGA microparticles, characterized by an initial burst followed by sustained release over 28 days, reflects typical polymer degradation and diffusion-controlled kinetics. This release behavior is consistent with established models of PLGA-based drug delivery, where initial surface-associated drug desorption precedes polymer erosion-mediated release [26,30]. The initial rapid release may facilitate early therapeutic concentrations, while the subsequent slow release maintains effective levels, a profile advantageous for bone healing applications that require both immediate and prolonged factor availability [31]. Comparatively, prior studies on ADPN receptor agonists have not addressed controlled release kinetics, limiting their translational application [32]. Moreover, the modulation of release rates by varying PLGA composition and molecular weight is well documented, suggesting avenues for tailoring release to specific clinical needs [26]. The release kinetics observed herein support the feasibility of using PLGA microparticles to achieve controlled ADPN delivery, potentially enhancing osteogenic outcomes through sustained receptor activation. Notably, the cumulative release reached approximately 55% over 28 days, leaving around 45% unreleased. The PLGA microparticles demonstrate a prolonged release duration, exceeding 28 days, offering significant advantages for clinical fractures requiring extended therapeutic coverage.

Enhanced biomechanical properties and improved microstructure parameters were presented following treatment with ADPN-MPs: catwalk indicated accelerated healing in the intervention groups, with the ADPN-MPs group exhibiting more stable healing rates compared to the ADPN group; three-point bending test indicated enhanced elasticity in the intervention groups; micro-CT identified better bone density, trabecular thickness in trabecular and bigger areas in cortical bone. This is supported by evidence that ADPN signaling influences bone remodeling by promoting osteoblast differentiation and inhibiting osteoclastogenesis via pathways such as Akt-GSK3β-Wnt and suppression of RANKL [33]. The improved mechanical strength observed correlates with increased bone matrix deposition and mineralization, consistent with our results in vivo findings [27]. Furthermore, the gait analysis underscores the clinical relevance of such biomaterial-based interventions, reflecting not only structural but also functional recovery. Thus, our data provide mechanistic and functional evidence supporting the therapeutic potential of ADPN-MPs in promoting fracture healing.

The limitations of this study should be acknowledged. First, while our in vivo model demonstrates enhanced fracture healing, the sample size was relatively small, which may restrict the generalizability of the findings and their applicability to broader populations. Second, the use of murine cell lines may not fully recapitulate the complexity of human ADPN biology due to species-specific differences in ADPN isoforms, receptor subtype expression, and signaling pathways. Third, this study did not include an empty PLGA microparticle control group; future studies incorporating such a control would help to clarify the baseline inflammatory response to the carrier matrix alone. Fourth, the high variance observed in the cumulative release profile is likely attributable to variable amounts of ADPN adsorbed onto the microparticle surface during the self-healing process. For future optimization, we may remove ADPN adsorbed on the surface of MPs via washing, then coat the outer surface of MPs with a membrane carrying charges opposite to ADPN, and re-adsorb ADPN to achieve stable external adsorption and internal loading, thereby yielding a more stable release profile [34].

## 5. Conclusions

In summary, this research successfully developed ADPN-MPs that preserved the ADPN structure and bioactivity. The formulation exhibited high loading efficiency, sustained release profile, and excellent biocompatibility. It promoted cell proliferation and supported bone healing to an extent comparable to free ADPN in several assays. By overcoming ADPN’s inherent instability, this PLGA-based delivery system offers a promising and translatable strategy for clinical bone repair applications.

## Figures and Tables

**Table 1:** The drug loading capacity and loading efficiency of adiponectin self-healing porous microparticles.

Groups	ADPN Before Drug Loading (μg)	ADPN After Drug Loading (μg)	Loaded ADPN (μg)	Total Mass (mg)	DL (%)	LE(%)
ADPN-MPs-1	2051.59	709.48	1342.11	148.16	0.91	65.42
ADPN-MPs-2	2051.59	536.13	1515.46	148.34	1.02	73.87
ADPN-MPs-3	2051.59	611.19	1440.40	148.26	0.97	70.21
Mean ± SD	2051.59 ± 0	618.93 ± 86.93	1432.66 ± 86.93	148.25 ± 0.09	0.97 ± 0.06	69.83 ± 4.24

Note: DL: Drug Loading, LE: Loading Efficiency, ADPN: Adiponectin, ADPN-MPs: Adiponectin self-healing microparticles.

**Table 2:** The particle size and distribution of MPs and ADPN-MPs.

Groups	D_10_ (μm)	D_50_ (μm)	D_90_ (μm)	Span
MPs	9.81	38.26	79.86	1.83
ADPN-MPs	3.83	30.35	67.29	2.09

Note: MPs: porous microparticles, ADPN-MPs: Adiponectin self-healing microparticles. Span = (D_90_ − D_10_)/D_50_. (Span is the dispersion coefficient; D_10_, D_50_, and D_90_ are the microparticle diameters measured at particle size ratios of 10%, 50%, and 90%, respectively).

## Data Availability

The original contributions presented in the study are included in the article, and further inquiries can be directed to the corresponding author.

## Supplementary Materials

The following supporting information can be downloaded at: https://www.mdpi.com/article/10.3390/pharmaceutics18050546/s1, Table S1. Information of the MC3T3-E1. Table S2. Information of the BMSCs.
