# Supplementary material for: PLGA Microparticles as a Stable and Biocompatible Carrier for Adiponectin Delivery to Enhance Bone Regeneration
Source: Pharmaceutics. 2026 Apr 29;18(5):546. doi: 10.3390/pharmaceutics18050546 (PMC13211176; doi:10.3390/pharmaceutics18050546)
Supplement: Supplementary file 1 [file pharmaceutics-18-00546-s001.zip › Supplementary Table S1.pdf]

Table S1. Information of the MC3T3-E1

**Item**	**MC3T3-E1**
1. Cell Type	Mouse pre-osteoblastic cell line (MC3T3-E1)
2. Specific Clone	Subclone 4
3. Source	Commercially purchased from the American Type Culture Collection (ATCC)
4. Catalog Number	CRL-2593™
5. Species/Strain	Mouse (*Mus musculus*), C57BL/6
6. Tissue of Origin	Calvaria
7. Cell Nature	Immortalized cell line
8. Key Characteristics	High capacity for osteogenic differentiation and extracellular matrix mineralization upon induction.
9. Culture Medium	α-MEM supplemented with 10% fetal bovine serum (FBS) and 1% penicillin-streptomycin
10. Quality Control	Cell line identity confirmed by STR profiling.<br>Routinely tested and confirmed negative for mycoplasma contamination.
11. Rationale for Selection	Subclone 4 is a well-established and widely used model for studying osteoblast differentiation and bone formation in vitro, known for its robust and reproducible osteogenic potential.
12. Ethical Approval	All procedures were approved by the Animal Ethics Committee of the Experimental Animal Center, Chinese PLA General Hospital (Approval No. 2019-X15-11).
