# Supplementary material for: PLGA Microparticles as a Stable and Biocompatible Carrier for Adiponectin Delivery to Enhance Bone Regeneration
Source: Pharmaceutics. 2026 Apr 29;18(5):546. doi: 10.3390/pharmaceutics18050546 (PMC13211176; doi:10.3390/pharmaceutics18050546)
Supplement: Supplementary file 1 [file pharmaceutics-18-00546-s001.zip › Supplementary Table S2.pdf]

Table S2. Information of the BMSCs

Item	BMSCs
1. Cell Type	Rat bone marrow-derived mesenchymal stem cells (BMSCs)
2. Species/Strain	3-day-old Sprague-Dawley (SD) rat pups
3. Tissue Source	Bone marrow from bilateral femurs
4. Cell Nature	Primary cells
5. Isolation Method	Bone marrow flushing of the femoral cavity
6. Culture Medium	α-MEM supplemented with 10% fetal bovine serum (FBS)
7. Culture Conditions	37°C in a humidified atmosphere containing 5% CO_2
8. Passaging Ratio	1:3
9. Characterization Method	Flow cytometry
10. Surface Marker Profile	Positive: CD90, CD29<br>Negative: CD34, CD45
11. Ethical Approval	All procedures were approved by the Animal Ethics Committee of the Experimental Animal Center, Chinese PLA General Hospital (Approval No. 2019-X15-11).
